# Supplementary material for: ALLM-Ab: Active Learning-Driven Antibody Optimization Using Fine-Tuned Protein Language Models
Source: J Chem Inf Model. 2025 Oct 22;65(21):11543–57. doi: 10.1021/acs.jcim.5c01577 (PMC12606632; doi:10.1021/acs.jcim.5c01577)
Supplement: Supplementary file 1 [file ci5c01577_si_001.pdf]

Supporting Information: ALLM-Ab: Active Learning-Driven Antibody Optimization Using Fine-tuned Protein Language Models

Kairi Furui and Masahito Ohue*

Department of Computer Science, School of Computing, Institute of Science Tokyo, Yokohama 226-8501, Japan

E-mail: ohue@comp.isct.ac.jp

Phone: +81 (0)45 924 5522. Fax: +81 (0)45 924 5523

Table S1: Configuration of LoRA target layers for each model.

Model	Target Modules
ProteinMPNN	W1, W2, W3, W11, W12, W13, W_in, W_out
ESM2	q_proj, k_proj, v_proj, out_proj, lm_head.dense
AbLang2	q_proj, k_proj, v_proj, out_proj, intermediate_layer.0, intermediate_layer.2

Table S2: Description of antibody developability metrics used in external evaluation. These metrics were calculated using Tamarind.[S6] Note that TAP uses values and thresholds obtained from Tamarind's reproduced implementation, which differ from the original by Raybould *et al.*[S1]

Metrics	Description
PSH	Patches of Surface Hydrophobicity. Must be within acceptable range. An indicator of the degree of surface hydrophobic patches near CDRs, where higher values indicate the presence of hydrophobic residues in close proximity. High values suggest risks of non-specific binding or high blood clearance.[S1]
PPC	Patches of Positive Charges.[S2] When too high, there are risks of non-specific binding or high blood clearance, and it needs to be less than 0.73.
PNC	Patches of Negative Charges.[S3] When too high, it can cause decreased expression levels or structural instability issues, and it needs to be less than 1.28.
SFvCSP	Structural Fv charge symmetry represents the asymmetry of surface charges between heavy and light chains.[S4,S5] Larger negative values increase the risk of viscosity increase, and it needs to be -1.59 or higher.
Heavy OASis Percentile	Heavy chain humanization score based on 9-mer peptide search in the Observed Antibody Space (OAS) calculated by BioPhi, where larger values indicate greater human-likeness.
SAPpos	Spatial aggregation propensity of CDR-H3 calculated by DeepSP, where larger values indicate more hydrophobic regions and higher aggregation risk.
SCMpos	Positive Spatial Charge Map of CDR-H3 calculated by DeepSP, where extremely high values pose aggregation risks.
SCMneg	Negative Spatial Charge Map of CDR-H3 calculated by DeepSP.

Table S3: Comparison of average time for sampling 100,000 sequences with and without applying approximation score.

Method	Execution Time (min)
Normal	17.0
Approx	1.12

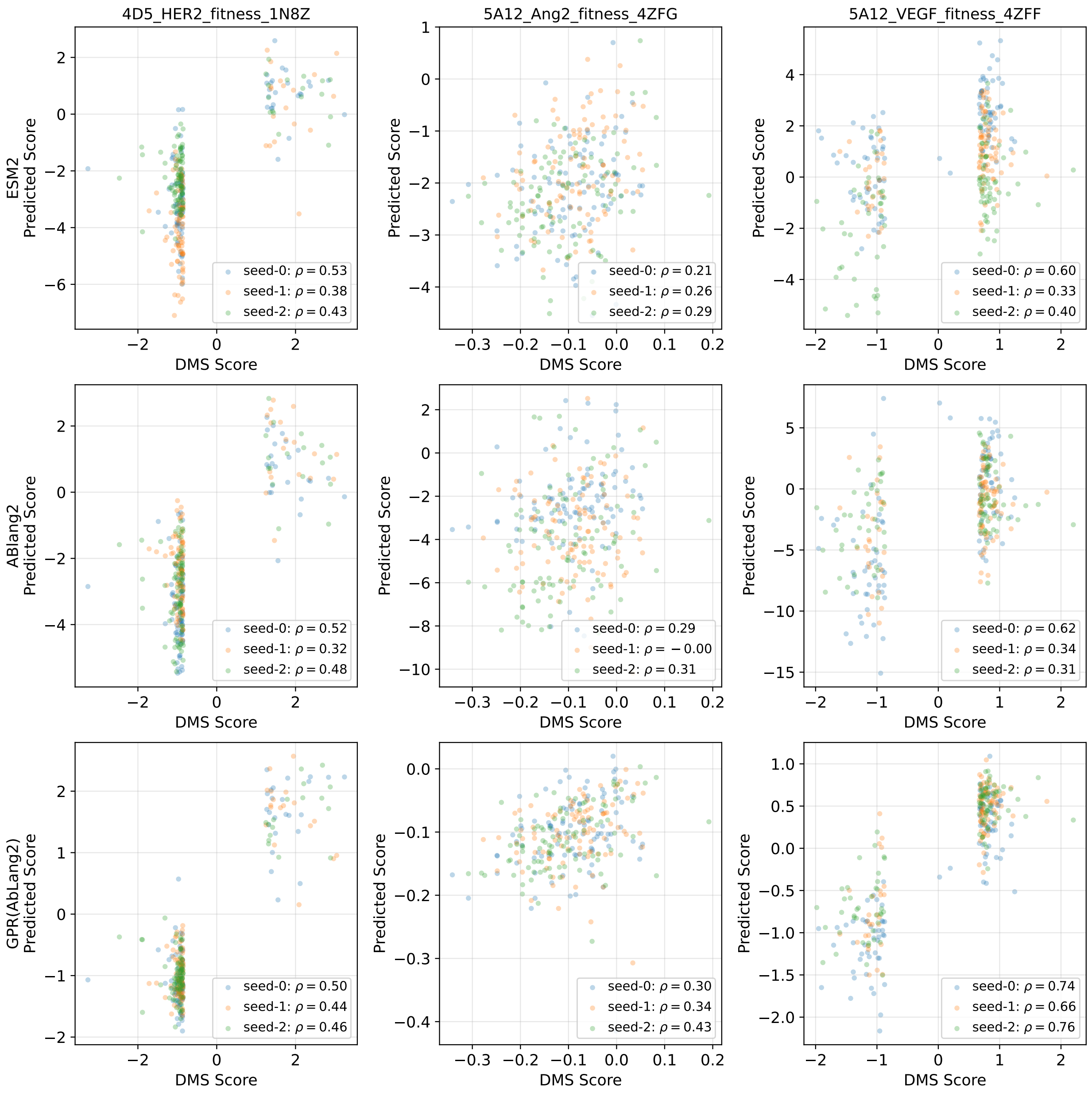

Figure S1: Scatter plots of DMS scores versus predicted scores on the test set for each target and model.

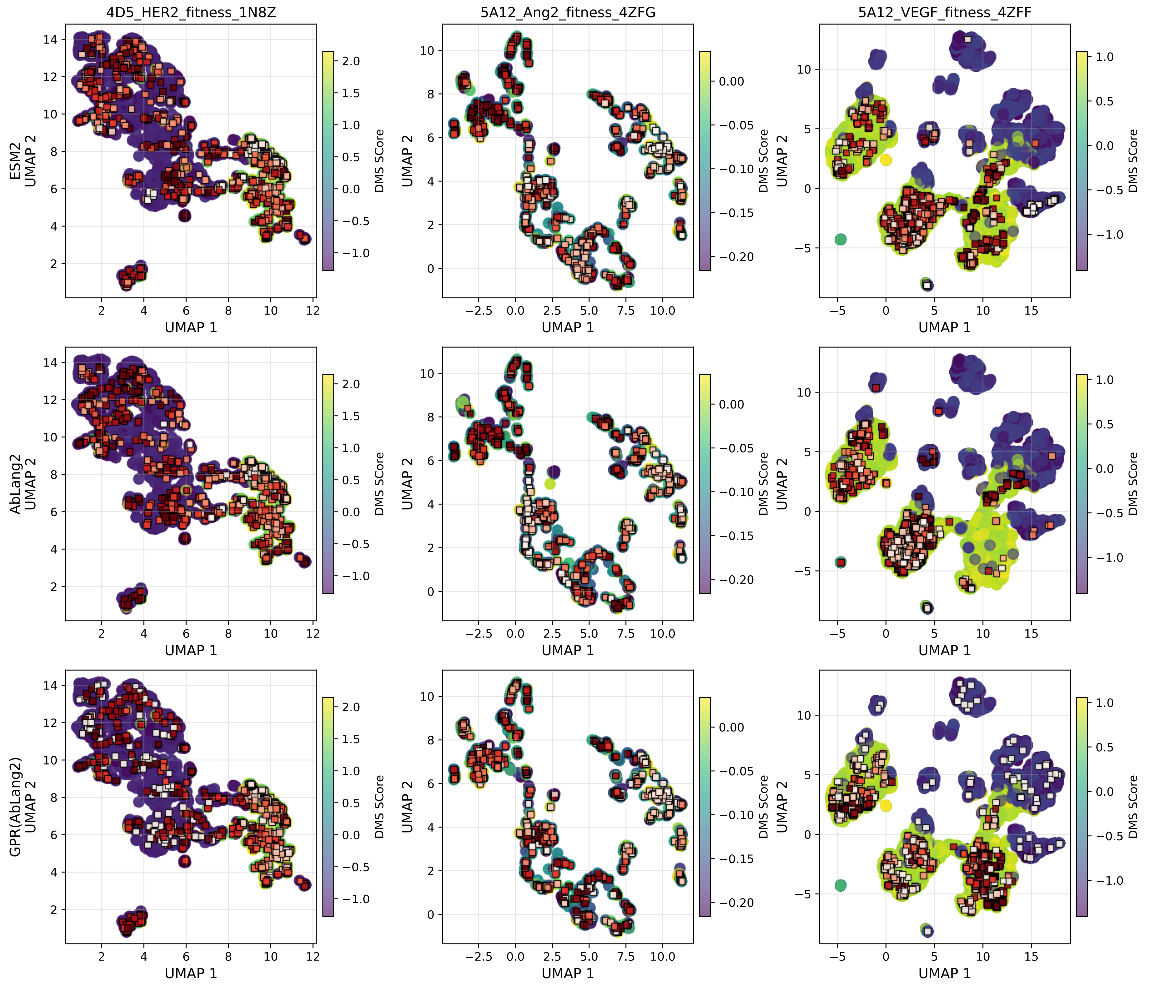

Figure S2: Distribution of variants selected in offline active learning experiments. This visualizes the distribution of AbLang2's 480-dimensional latent variables reduced to 2 dimensions using UMAP. Square points represent variants selected in online active learning experiments, with redder colors indicating selection in later cycles.

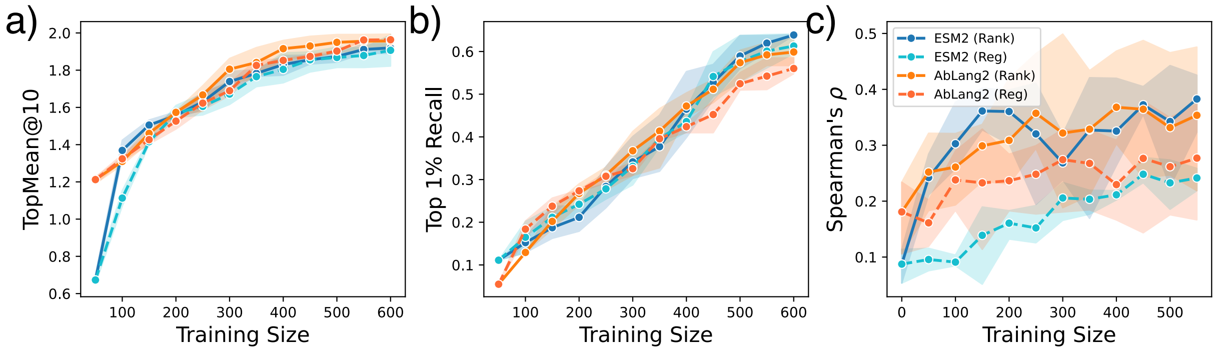

Figure S3: Comparison of active learning performance when using learning-to-rank (ListMLE loss) versus regression learning (MSE loss). (a) TopMean@10, (b) Top 1% Recall, (c) Spearman's ρ.

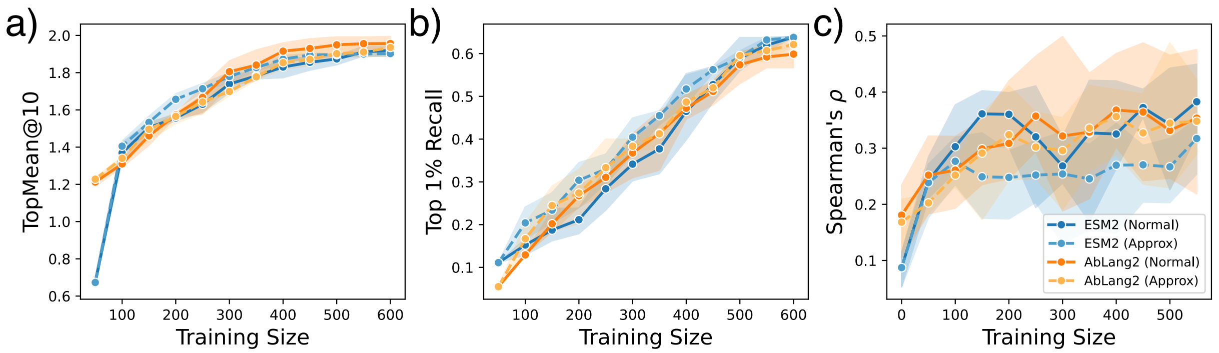

Figure S4: Comparison of active learning performance when using the fitness score (normal mode) versus the approximation score (approx mode). (a) TopMean@10, (b) Top 1% Recall, (c) Spearman's ρ.

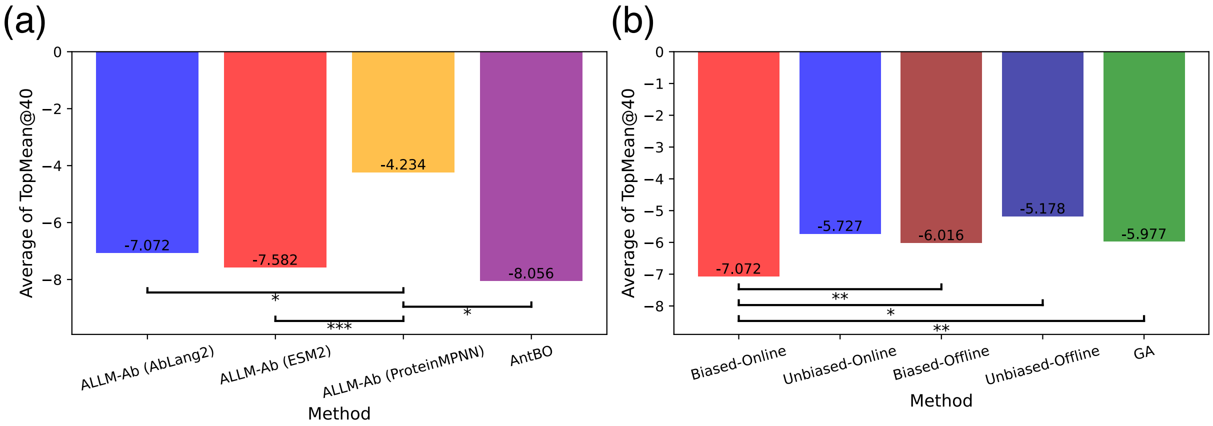

Figure S5: Average TopMean@40 values for each target in the final cycle of single-objective optimization experiments. (a) Comparison of models. (b) Comparison of mutant generation methods. * indicates difference at 5% significance level, ** indicates difference at 1% significance level by Wilcoxon signed-rank test.

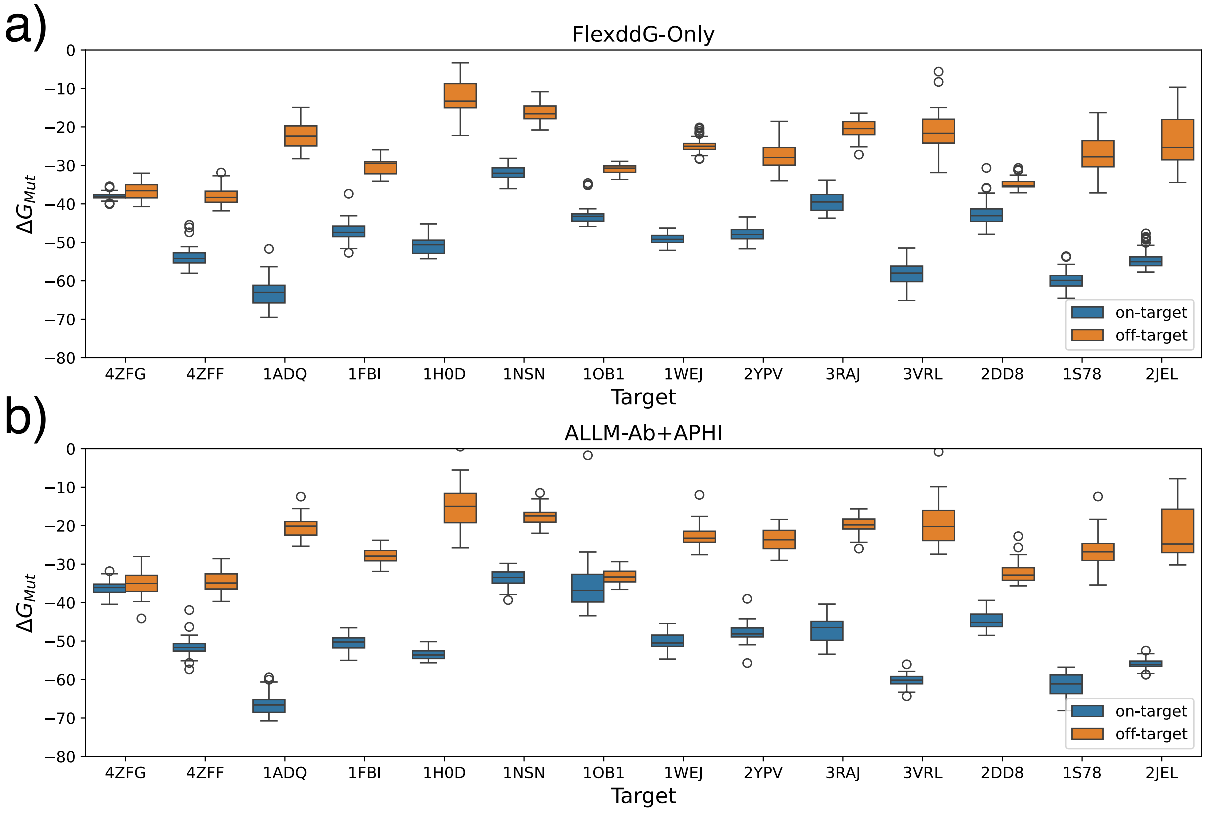

Figure S6: Evaluation of specificity using binding free energy ΔG_{mut} of mutants calculated by FlexddG in single-objective optimization experiments. (a) FlexddG-Only, (b) ALLM-Ab+APHI. On-target represents ΔG_{mut} for the target antigen, while Off-target represents ΔG_{mut} calculated from the predicted complex structure between HER2(PDB:1N8Z) and antibody. Using HER2(PDB:1N8Z) as an off-target, complex structures were predicted with Boltz-2,[S7,S8] and the 1N8Z complex pose was reproduced using potential-enhanced templates.

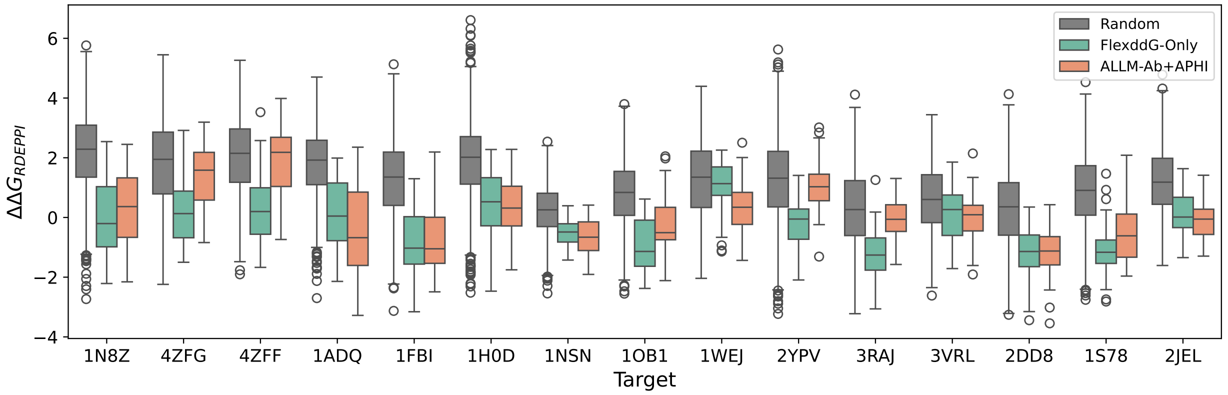

Figure S7: External validation of binding affinity improvement using the state-of-the-art deep learning-based $\Delta\Delta G$ prediction model RDE-PPI. Results when predicting $\Delta\Delta G$ with RDE-PPI for random mutants from test data and mutants finally selected by FlexddG-Only and ALLM-Ab+APHI. Compared to random mutants, the predicted $\Delta\Delta G$ values for mutants explored by the two methods are lower, confirming that FlexddG computational values correlate with external evaluation.

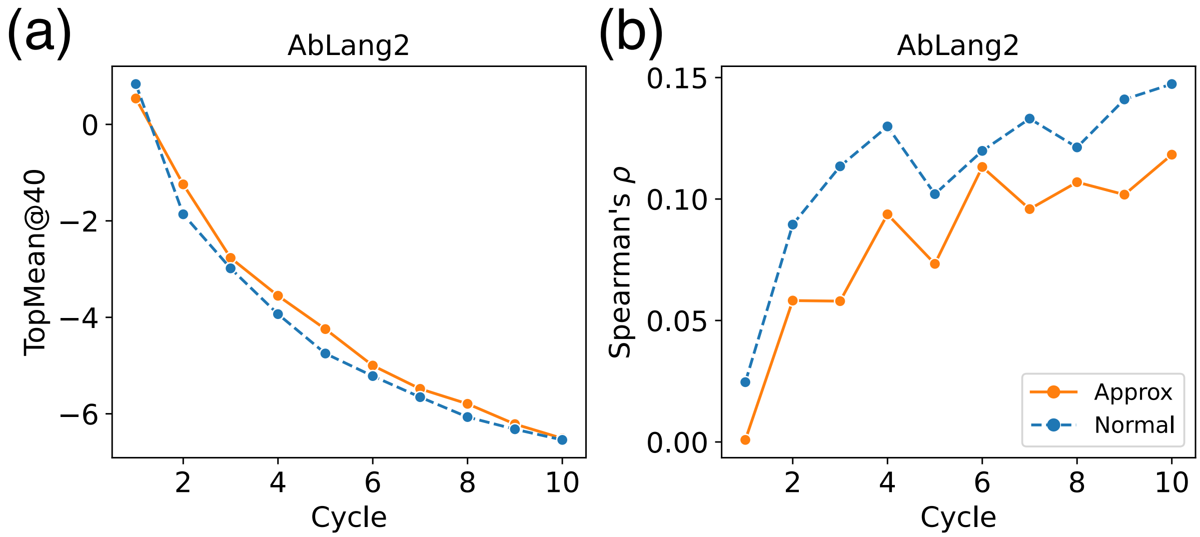

Figure S8: Evolution of TopMean@40 of $\Delta\Delta G_{FlexddG}$ and Spearman's ρ when using fitness score (normal mode) versus approximation score (approx mode) in ALLM-Ab. (a) TopMean@40, (b) Spearman's ρ.

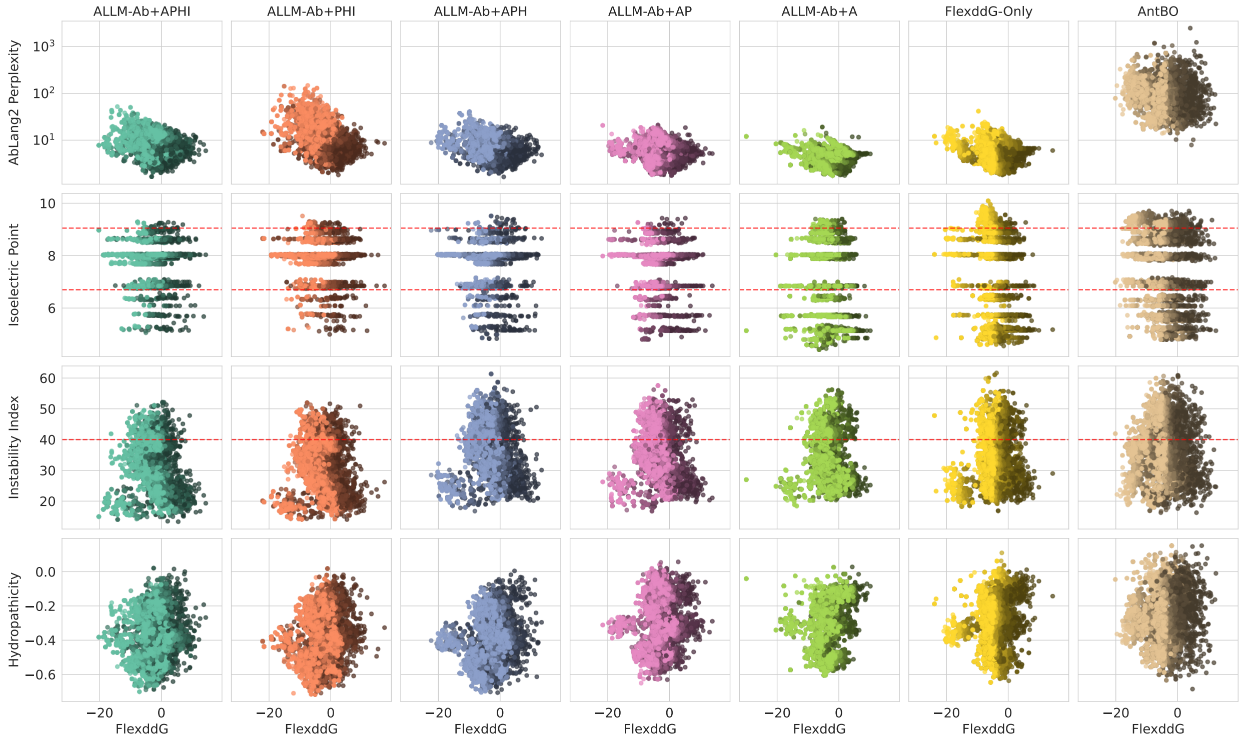

Figure S9: Scatter plots showing the evolution of Flex ddG and developability metrics for the top 40 mutants selected by each multiobjective optimization method in each cycle. Brighter colored points represent mutants that were ranked highly in later cycles.

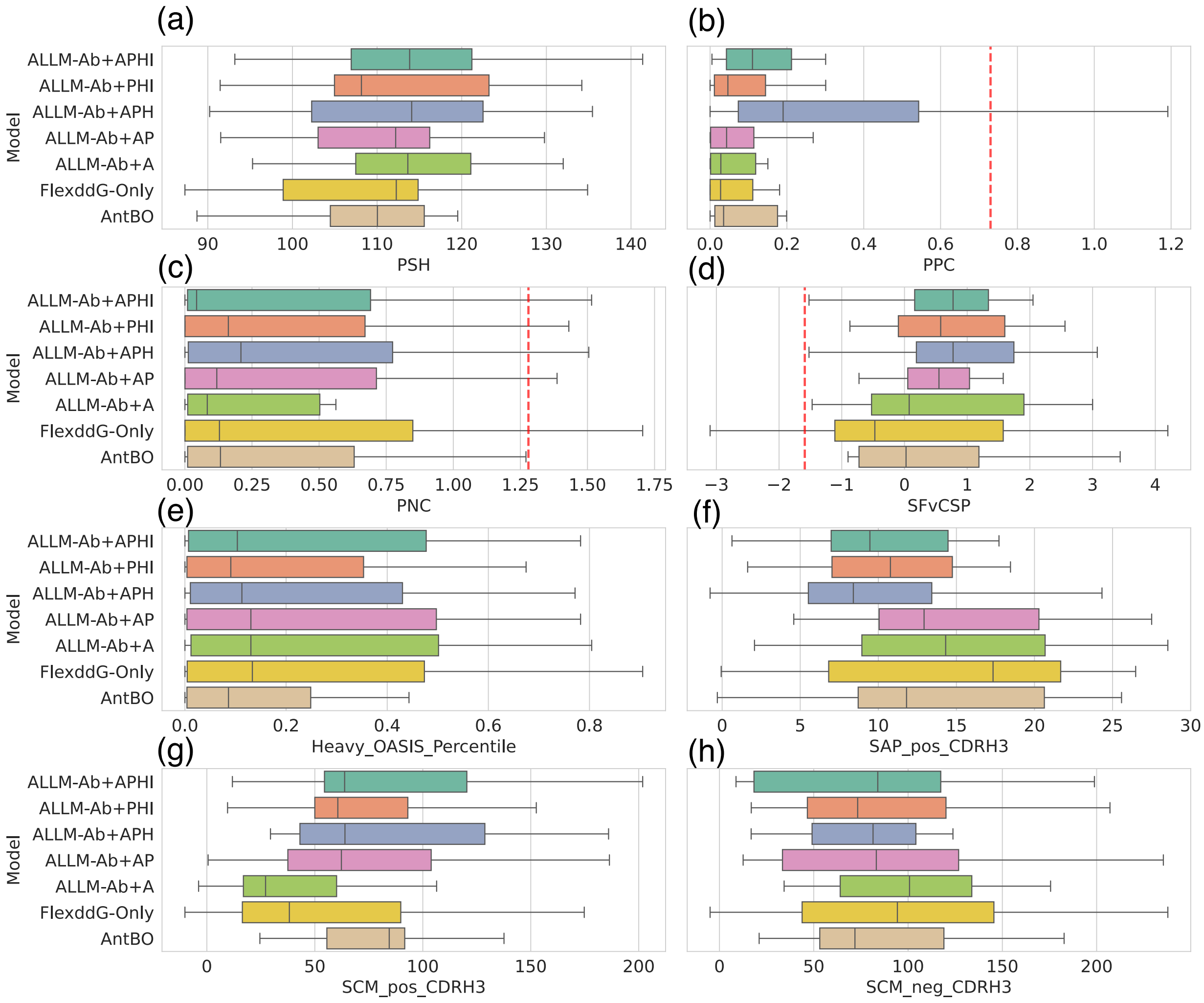

Figure S10: Box plots showing the distribution of antibody developability metrics for the top 1 mutant from each multiobjective optimization method. (a) PSH, (b) PPC, (c) PNC, (d) SFvCSP, (e) Heavy OASis Percentile, (f) `SAP_pos_CDR-H3`, (g) `SCM_pos_CDR-H3`, (h) `SCM_neg_CDR-H3`.

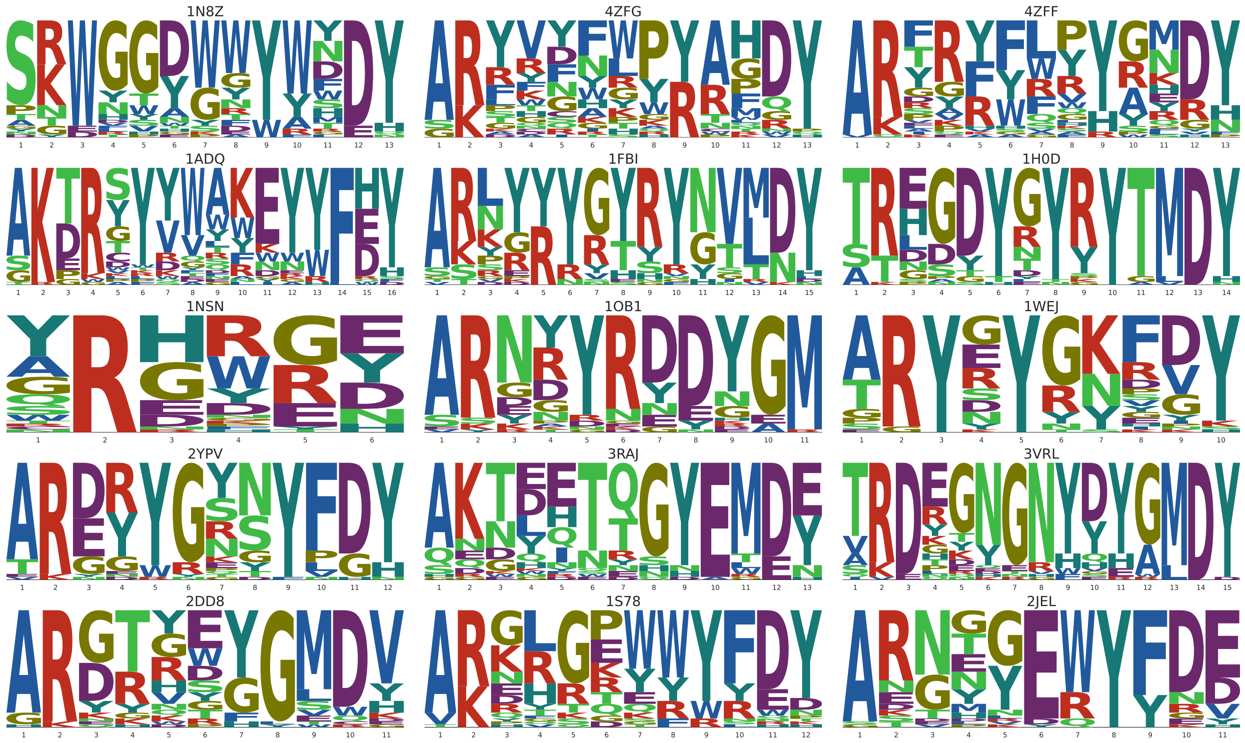

Figure S11: Sequence logos of the top 40 mutants selected by ALLM-Ab+APHI for 15 targets.

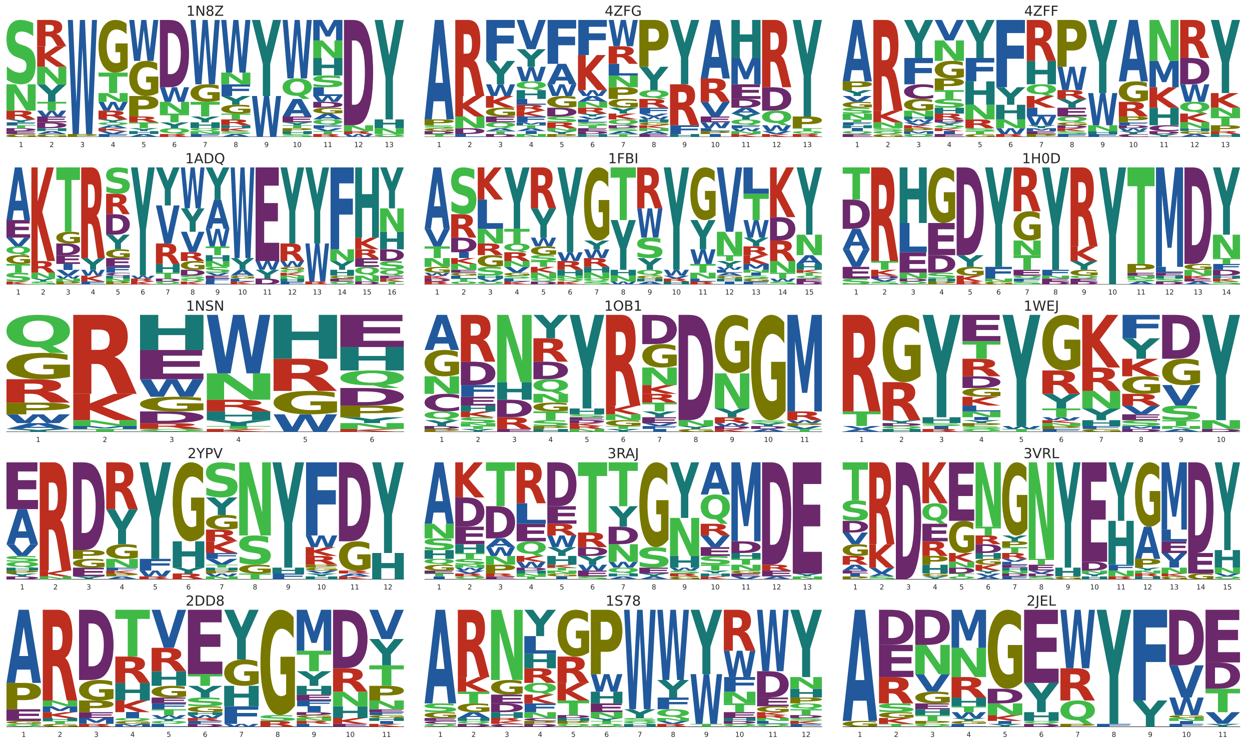

Figure S12: Sequence logos of the top 40 mutants selected by ALLM-Ab+PHI for 15 targets.

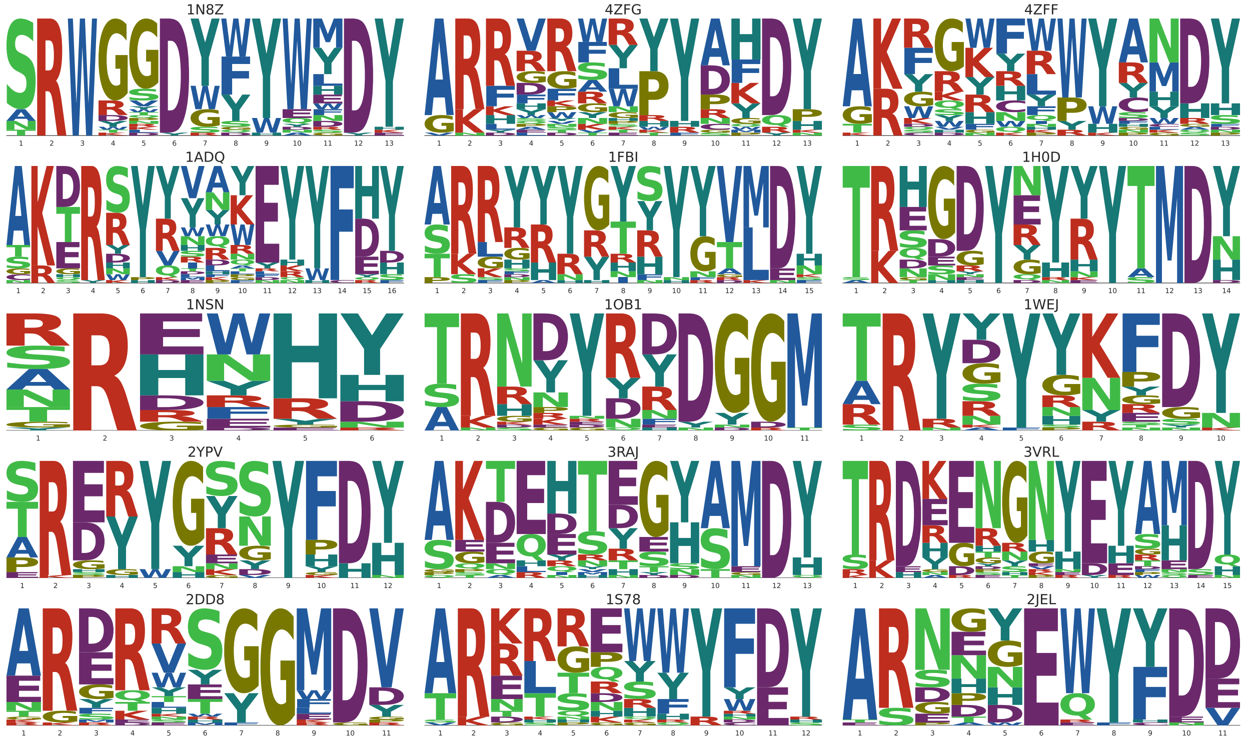

Figure S13: Sequence logos of the top 40 mutants selected by ALLM-Ab+APH for 15 targets.

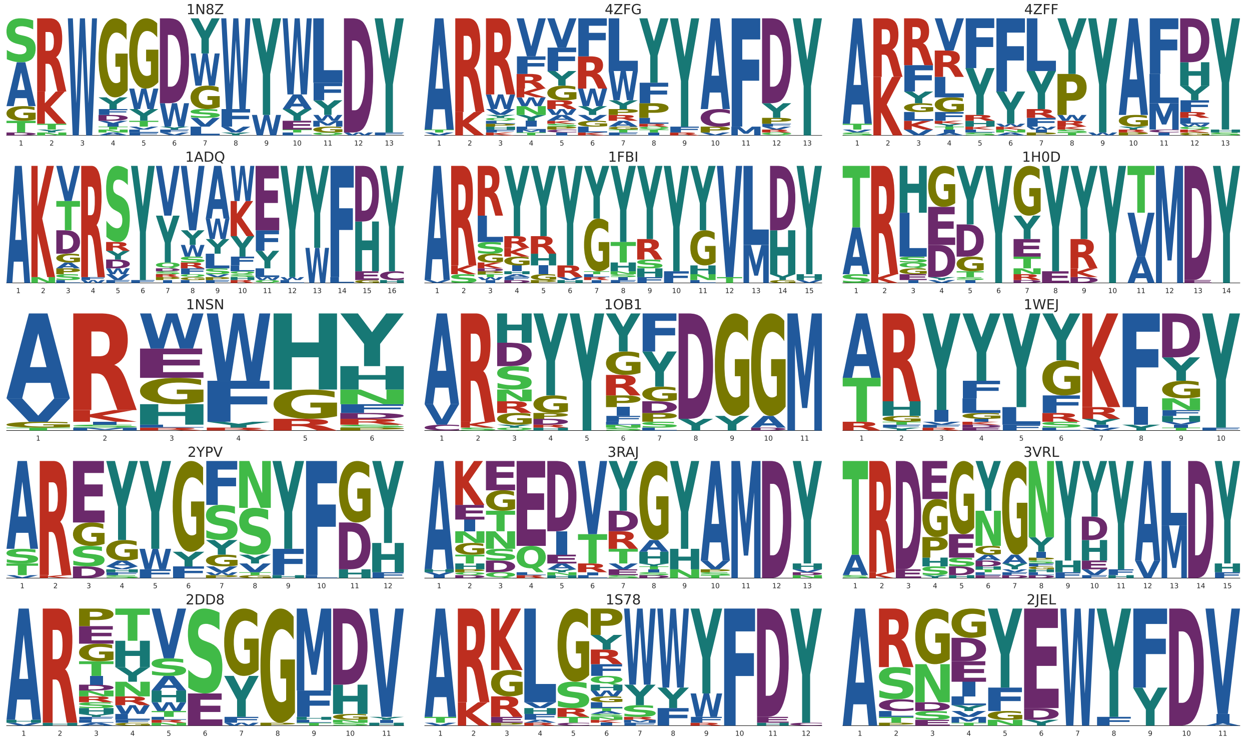

Figure S14: Sequence logos of the top 40 mutants selected by ALLM-Ab+AP for 15 targets.

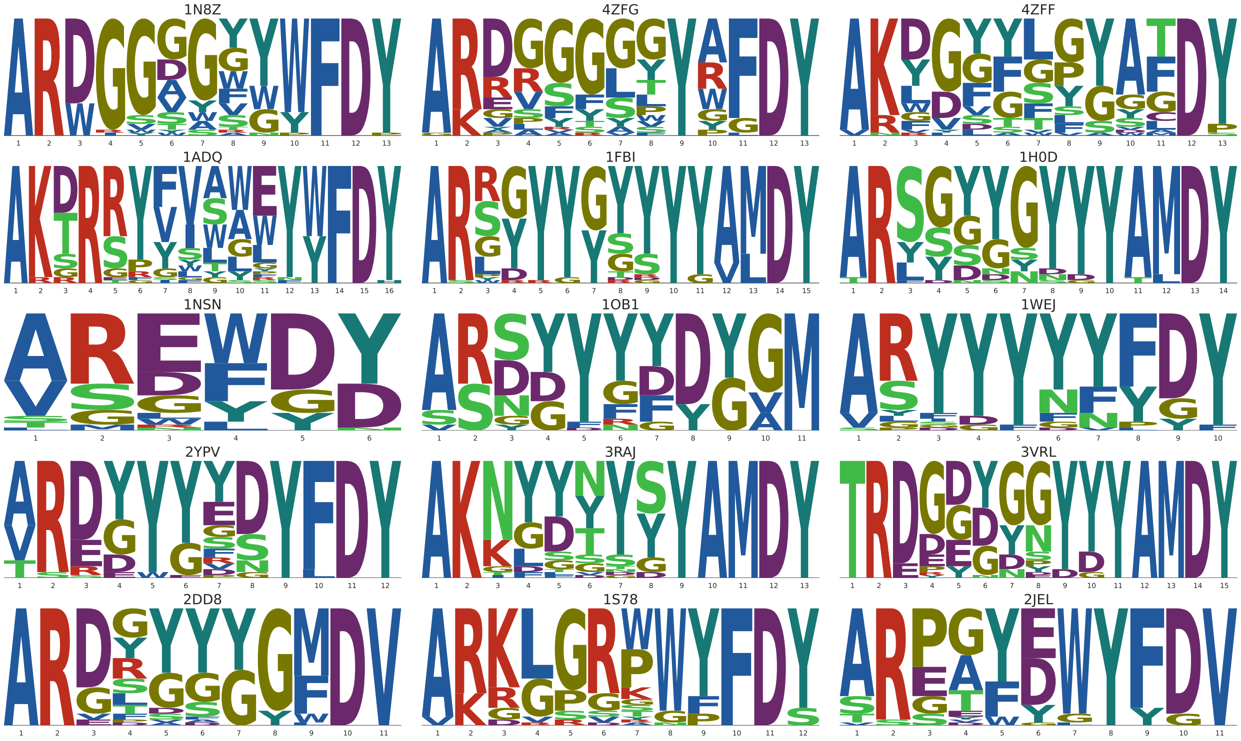

Figure S15: Sequence logos of the top 40 mutants selected by ALLM-Ab+A for 15 targets.

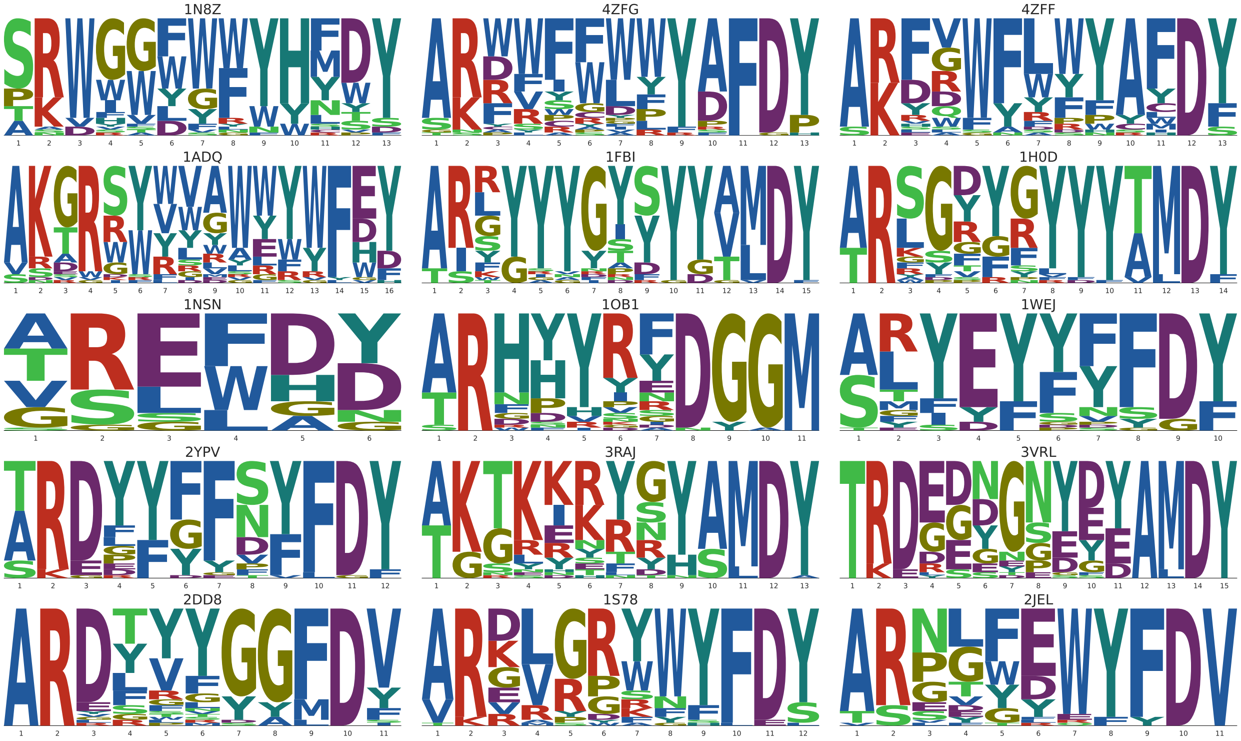

Figure S16: Sequence logos of the top 40 mutants selected by FlexddG-Only for 15 targets.

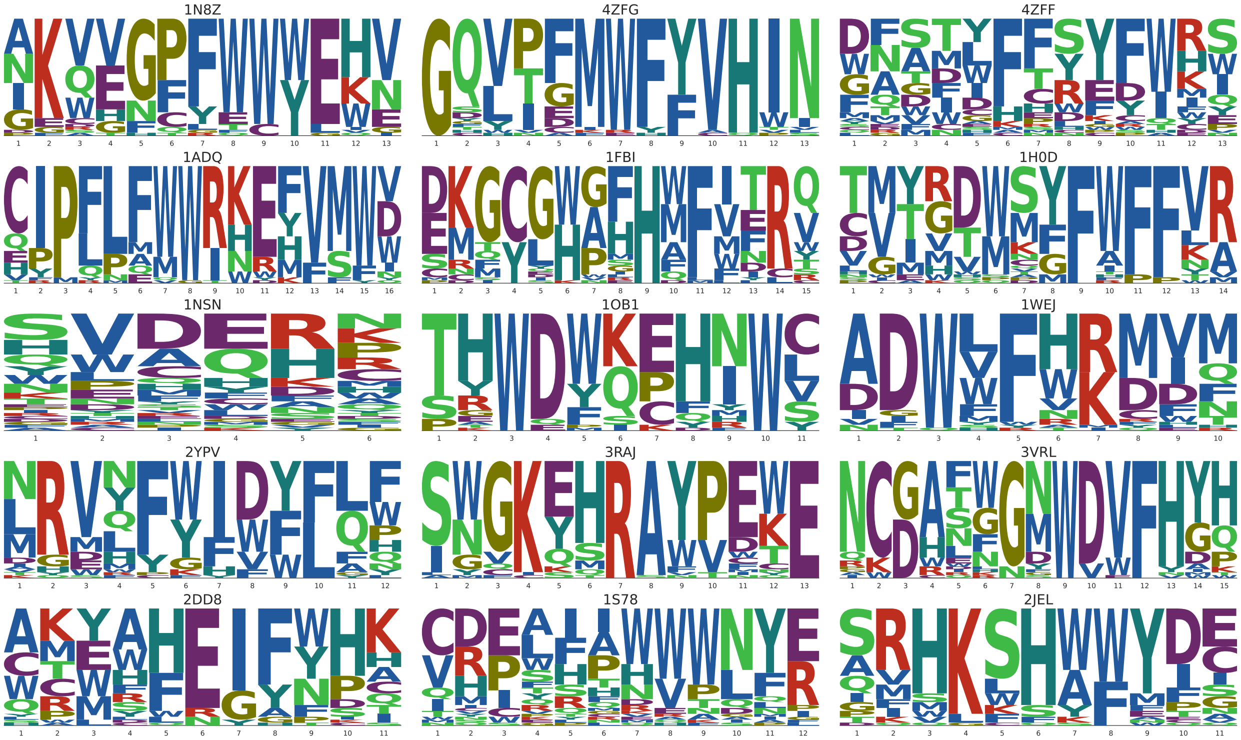

Figure S17: Sequence logos of the top 40 mutants selected by AntBO for 15 targets.

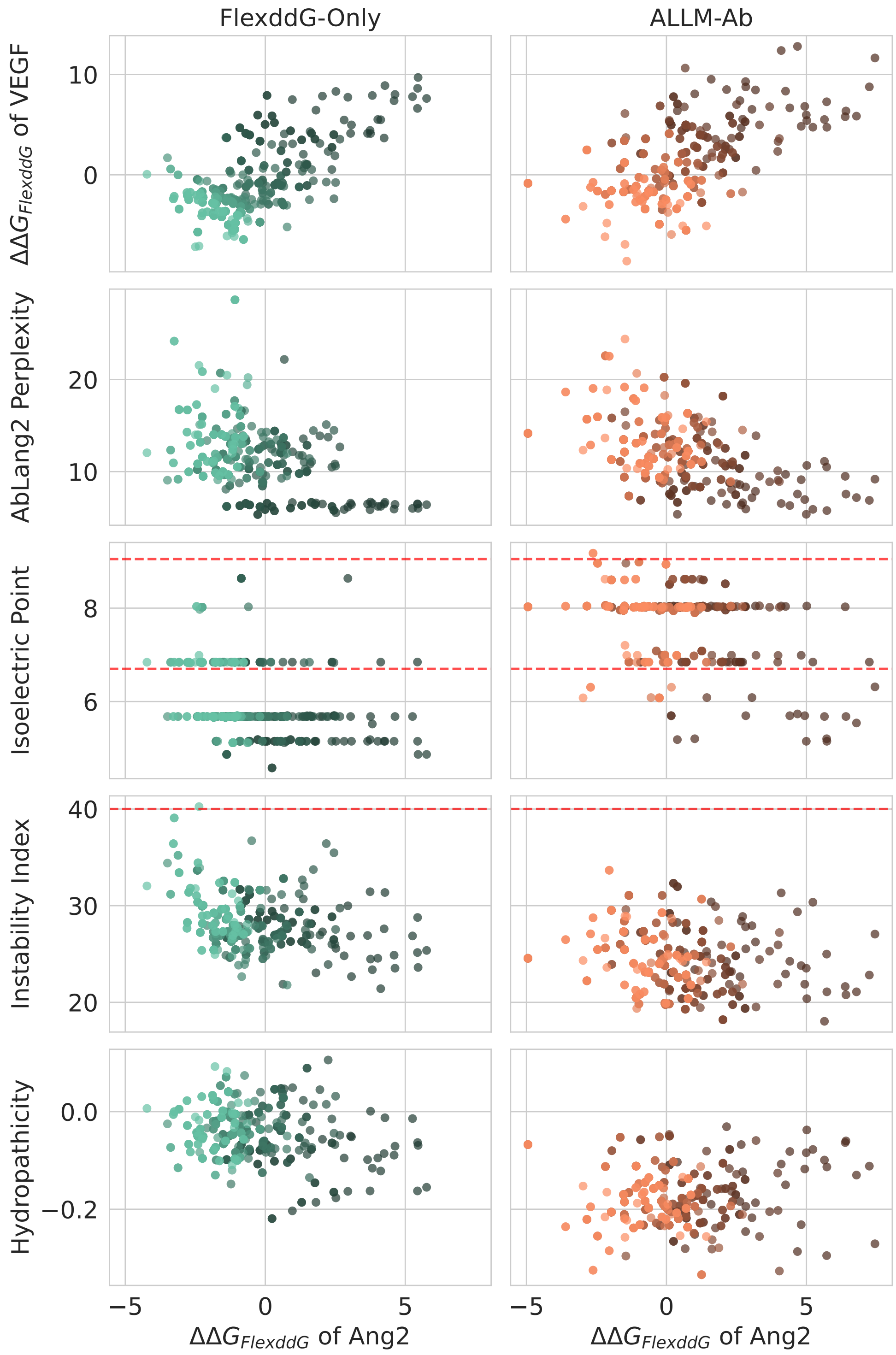

Figure S18: Scatter plots showing the evolution of Flex ddG and developability metrics for the top 40 mutants selected by dual optimization in each cycle. (a) FlexddG-Only, (b) ALLM-Ab. Brighter colored points represent mutants that were ranked highly in later cycles.

References

(S1) Raybould, M. I. J.; Marks, C.; Krawczyk, K.; Taddese, B.; Nowak, J.; Lewis, A. P.; Bujotzek, A.; Shi, J.; Deane, C. M. Five computational developability guidelines for therapeutic antibody profiling. *Proc. Natl. Acad. Sci. U. S. A.* **2019**, *116*, 4025–4030.

(S2) Datta-Mannan, A.; Thangaraju, A.; Leung, D.; Tang, Y.; Witcher, D. R.; Lu, J.; Wroblewski, V. J. Balancing charge in the complementarity-determining regions of humanized mAbs without affecting pI reduces non-specific binding and improves the pharmacokinetics. *mAbs* **2015**, *7*, 483–493.

(S3) Popovic, B.; Gibson, S.; Senussi, T.; Carmen, S.; Kidd, S.; Slidel, T.; Strickland, I.; Jianqing, X.; Spooner, J.; Lewis, A.; Hudson, N.; Mackenzie, L.; Keen, J.; Kemp, B.; Hardman, C.; Hatton, D.; Wilkinson, T.; Vaughan, T.; Lowe, D. Engineering the expression of an anti-interleukin-13 antibody through rational design and mutagenesis. *Protein Eng. Des. Sel.* **2017**, *30*, 303–311.

(S4) Sharma, V. K.; Patapoff, T. W.; Kabakoff, B.; Pai, S.; Hilario, E.; Zhang, B.; Li, C.; Borisov, O.; Kelley, R. F.; Chorny, I.; Zhou, J. Z.; Dill, K. A.; Swartz, T. E. In silico selection of therapeutic antibodies for development: viscosity, clearance, and chemical stability. *Proc. Natl. Acad. Sci. U. S. A.* **2014**, *111*, 18601–18606.

(S5) Yadav, S.; Laue, T. M.; Kalonia, D. S.; Singh, S. N.; Shire, S. J. The influence of charge distribution on self-association and viscosity behavior of monoclonal antibody solutions. *Mol. Pharm.* **2012**, *9*, 791–802.

(S6) Tamarind Bio State of the art computational tools for biology. `https://www.tamarind.bio/`, 2024; Accessed: 17 October, 2025.

(S7) Wohlwend, J.; Corso, G.; Passaro, S.; Reveiz, M.; Leidal, K.; Swiderski, W.; Portnoi, T.; Chinn, I.; Silterra, J.; Jaakkola, T.; Barzilay, R. Boltz-1 democratizing biomolecular interaction modeling. *bioRxiv* **2024**, 2024.11.19.624167.

(S8) Passaro, S.; Corso, G.; Wohlwend, J.; Reveiz, M.; Thaler, S.; Somnath, V. R.; Getz, N.; Portnoi, T.; Roy, J.; Stark, H.; Kwabi-Addo, D.; Beaini, D.; Jaakkola, T.; Barzilay, R. Boltz-2: Towards accurate and efficient binding affinity prediction. *bioRxiv* **2025**, 2025.06.14.659707.
